# How do gravity alterations affect animal and human systems at a cellular/tissue level?

**DOI:** 10.1038/s41526-023-00330-y

**Published:** 2023-10-21

**Authors:** Francesca Cialdai, Austin M. Brown, Cory W. Baumann, Debora Angeloni, Sarah Baatout, Alexandra Benchoua, Juergen Bereiter-Hahn, Daniele Bottai, Judith-Irina Buchheim, Marco Calvaruso, Eugénie Carnero-Diaz, Sara Castiglioni, Duccio Cavalieri, Gabriele Ceccarelli, Alexander Choukér, Gianni Ciofani, Giuseppe Coppola, Gabriella Cusella, Andrea Degl’Innocenti, Jean-Francois Desaphy, Jean-Pol Frippiat, Michael Gelinsky, Giada Genchi, Maria Grano, Daniela Grimm, Alain Guignandon, Christiane Hahn, Jason Hatton, Raúl Herranz, Christine E. Hellweg, Carlo Saverio Iorio, Thodoris Karapantsios, Jack van Loon, Matteo Lulli, Jeanette Maier, Jos Malda, Emina Mamaca, Lucia Morbidelli, Angelique van Ombergen, Andreas Osterman, Aleksandr Ovsianikov, Francesco Pampaloni, Elizabeth Pavezlorie, Veronica Pereda-Campos, Cyrille Przybyla, Christopher Puhl, Petra Rettberg, Chiara Risaliti, Angela Maria Rizzo, Kate Robson-Brown, Leonardo Rossi, Giorgio Russo, Alessandra Salvetti, Daniela Santucci, Matthias Sperl, Felice Strollo, Kevin Tabury, Sara Tavella, Christiane Thielemann, Ronnie Willaert, Nathaniel J. Szewczyk, Monica Monici

**Affiliations:** 1 ASAcampus Joint Laboratory, ASA Res. Div., DSBSC-University of Florence, Florence, Italy; 2 Honors Tutorial College, Ohio University, Athens, OH USA; 3 Heritage College of Osteopathic Medicine, Ohio University, Athens, OH USA; 4 Inst. of Biorobotics, Scuola Superiore Sant’Anna, Pisa, Italy; 5 Radiobiology Unit, Belgian Nuclear Research Centre (SCK CEN) Boeretang 200, 2400 Mol, Belgium; 6 ISTEM, CECS, AFM-Téléthon, Corbeil-Essonnes, France; 7 Inst. for Cell and Neurobiol, Goethe University Frankfurt am Main, Frankfurt am Main, Germany; 8 Dept. Pharmaceutical Sciences, University of Milan, Milan, Italy; 9 Laboratory of “Translational Research, Stress & Immunity”, Department of Anesthesiology, LMU University Hospital Munich, Munich, Germany; 10 Inst. Molecular Bioimaging and Physiology, National Research Council (IBFM-CNR), Cefalù, Italy; 11 Inst. Systematic, Evolution, Biodiversity, Sorbonne University, NMNH, CNRS, EPHE, UA, Paris, France; 12 Dept. of Biomedical and Clinical Sciences, University of Milan, Milan, Italy; 13 Dept. of Biology, University of Florence, Florence, Italy; 14 Dept of Public Health, Experimental Medicine and Forensic, University of Pavia, Pavia, Italy; 15 Smart Bio-Interfaces, Istituto Italiano di Tecnologia, 56025 Pontedera (PI), Italy; 16 Institute of Applied Science and Intelligent Sistems – CNR, Naples, Italy; 17 Dept Medical Biotechnologies, University of Siena, Siena, Italy; 18 Smart Bio-Interfaces, IIT, Pontedera (PI), Italy; 19 Dept. Precision and Regenerative Medicine, University of Bari “Aldo Moro”, Bari, Italy; 20 Stress, Immunity, Pathogens Laboratory, SIMPA, Université de Lorraine, Nancy, France; 21 Centre for Translational Bone, Joint & Soft Tissue Research, TU Dresden, Dresden, Germany; 22 Dept. Microgravity and Translational Regenerative Medicine, Otto-von-Guericke-University Magdeburg, Magdeburg, Germany; 23 Dept of Biomedicine, Aarhus University, Aarhus, Denmark; 24 SAINBIOSE, INSERM U1059, Université Jean Monnet, F-42000 Saint-Etienne, France; 25 European Space Agency, Paris, France; 26 Centro de Investigaciones Biológicas Margarita Salas (CSIC), Madrid, Spain; 27 Radiation Biology Dept., Inst. of Aerospace Medicine, German Aerospace Center (DLR), Cologne, Germany; 28 CREST-ATM, Université libre de Bruxelles, Bruxelles, Belgium; 29 Faculty of Chemistry, Aristotle Univeristy of Thessaloniki, Thessaloniki, Greece; 30 Amsterdam University Medical Center, ACTA/VU, Amsterdam, The Netherlands; 31 Dept. Experimental and Clinical Biomedical Sciences, University of Florence, Florence, Italy; 32 Dept. Orthopaedics, Univ. Med. Center Utrecht & Dept. Clinical Sciences, Utrecht Univ, Utrecht, The Netherlands; 33 European and International Affairs Dept, Ifremer centre Bretagne, Plouzané, France; 34 Dept. Life Sciences, Univ. of Siena, Siena, Italy; 35 Max von Pettenkofer Institute, Virology, LMU Munich & DZIF, Partner Site Munich, Munich, Germany; 36 3D Printing and Biofabrication, Inst. Materials Science and Technology, TU Wien, Vienna, Austria; 37 Buchmann Inst. for Molecular Life Sciences, Goethe-Universität Frankfurt am Main, Frankfurt am Main, Germany; 38 Ludwig Boltzmann Inst. for Traumatology, Res. Center in Cooperation with AUVA, Vienna, Austria; 39 GSBMS/URU EVOLSAN - Medecine Evolutive, Université Paul Sabatier Toulouse III, Toulouse, France; 40 MARBEC, Univ Montpellier, CNRS, Ifremer, IRD, Palavas les Flots, France; 41 Space Applications NV/SA for European Space Agency, Houston, USA; 42 DLR, Inst of Aerospace Medicine, Research Group Astrobiology, Köln, Germany; 43 Dept. of Pharmacological and Biomolecular Sciences, University of Milan, Milan, Italy; 44 Dept of Engineering Mathematics, and Dept of Anthropology and Archaeology, University of Bristol, Bristol, UK; 45 Dept. Clinical and Experimental Medicine, University of Pisa, Pisa, Italy; 46 Center for Behavioural Sciences and Mental Health, Ist. Superiore Sanità, Rome, Italy; 47 DLR-MP, Cologne, Germany; 48 Endocrinology and Metabolism Unit, IRCCS San Raffaele Pisana, Rome, Italy; 49 IRCCS Ospedale Policlinico San Martino and University of Genoa, DIMES, Genoa, Italy; 50 BioMEMS lab, University of Applied Sciences Aschaffenburg, Aschaffenburg, Germany; 51 Research Group NAMI and NANO, Vrije Universiteit Brussels, Brussels, Belgium

**Keywords:** Medical research, Systems biology

## Abstract

The present white paper concerns the indications and recommendations of the SciSpacE Science Community to make progress in filling the gaps of knowledge that prevent us from answering the question: “How Do Gravity Alterations Affect Animal and Human Systems at a Cellular/Tissue Level?” This is one of the five major scientific issues of the ESA roadmap “Biology in Space and Analogue Environments”. Despite the many studies conducted so far on spaceflight adaptation mechanisms and related pathophysiological alterations observed in astronauts, we are not yet able to elaborate a synthetic integrated model of the many changes occurring at different system and functional levels. Consequently, it is difficult to develop credible models for predicting long-term consequences of human adaptation to the space environment, as well as to implement medical support plans for long-term missions and a strategy for preventing the possible health risks due to prolonged exposure to spaceflight beyond the low Earth orbit (LEO). The research activities suggested by the scientific community have the aim to overcome these problems by striving to connect biological and physiological aspects in a more holistic view of space adaptation effects.

## Introduction

Several undesirable consequences of space flight have been described since the very beginning of space exploration, including: bone loss, muscle atrophy, immune response impairment, nervous system functional derangement, cardiovascular deconditioning, metabolic dysregulation, spaceflight associated neuro-ocular syndrome (SANS) etc.^1–8^.

All of these alterations represent models of chronic diseases commonly observed on Earth, mostly affecting the elderly, and potentially sharing the same pathophysiological mechanism(s), involving oxidative stress and vascular dysfunction through chronic inflammation^9–14^. In addition, stressful tasks and diet changes, confinement-related artificial light, isolation and reduced motor activity might worsen the effects of weightlessness by further contributing to musculoskeletal and cardiovascular deconditioning, dysregulation of energy metabolism and endocrine balance^14–21^.

Despite the continuously increasing knowledge gathered on mechanisms underlying the multiple effects of space flight on astronauts, studies conducted so far have not been able to generate a synthetic integrated view of changes occurring at different system and functional levels. Therefore, it remains difficult to elaborate credible models predicting long-term consequences of human adaptation to the space environment, as well as, to plan the medical support and the necessary countermeasures for long-term missions.

Recent research progress points to new approaches to understand the inner mechanisms leading from health to the onset of pathological conditions. The ability to maintain/restore homeostasis is of critical importance to allow tissues to restore their functions and e.g. to heal from injuries and diseases allows the organism to survive. In contrast, persistence of altered homeostatic conditions and here the lack of e.g., efficient healing ability predispose to the onset of chronic diseases, accelerated aging, disability and death^22–25^.

Most recent research suggests the importance to study the connections among oxidative stress, increased endotoxin levels, metabolic dysregulation (MetS), low grade inflammation and immune system changes, which in turn may be related to altered microbiome/virome expressions^6,20,26–37^. A case study carried out on one subject engaged in a one-year mission has recently reported that alterations of the inflammatory, metabolic and immune profiles are among the more persistent alterations after return to Earth, with an early peak and a slow decreasing trend thereafter without coming back to pre-flight levels for months^28^. In other studies, a stress related shift toward inflammageing has been observed in cosmonauts after long-duration spaceflight^38^. Therefore, the microbiome-immune profile-inflammation-metabolism (including mitochondrial dysfunction and oxidative stress) seems to play a central role in adaptation mechanisms to spaceflight.

## Key knowledge gaps

To plan long-lasting deep space missions, the effects of altered gravity on animal and human systems (in particular the immune, musculoskeletal, cardiovascular, neurosensory, neuroendocrine, excretory, respiratory, metabolic and integumentary systems) should be further explored, focusing on molecular/cellular and cell-communication processes. These effects should be framed in the more general context of systemic adaptation to the space environment and contemporary alterations in different organs and functions, which can be induced also by factors different from altered gravity (e.g. radiation, isolation, confinement, celestial dust)^18,39,40^.

Efforts should be made to untangle the confounding effects of multiple stressors in the space environment (the space exposome), and differentiate the effects of space-related stressors (altered gravity and cosmic rays from the effects of confinement, isolation and psychophysical stress). In order to provide appropriate countermeasures, it is extremely important to clarify the causes and contributing factors of the various possible alterations.

It is mandatory to study the evolution of fundamental physiological processes of adaptation, crucial for organism’s survival, (e.g. hemorrhage and blood coagulation, acute and chronic inflammation, stromal activation, tissue repair mechanisms, metabolic pathways) in altered gravity, in order to predict long-term alterations and implement countermeasures. In fact, long-duration space exploration missions might reduce the organism’s ability to respond to acute injuries/infections and predispose astronauts to the onset of chronic diseases (e.g. cardiovascular and metabolic diseases) and premature aging by strongly influencing redox and metabolic processes, microbiota, immune function, as well as, endotoxin and pro-inflammatory signal production^6,21,28,31–35,37,41–44^. Indeed, it is known that low grade chronic inflammation (LGI) involves many of the above mechanisms and is a distinctive feature of major chronic diseases in the aging population^45–47^.

Moreover, we need to know the long-term consequences of the above effects. In other words, we need to know if and how changes in the crucial biological processes mentioned above can increase the risk of acute and chronic disease onset and/or influence disease progression.

In summary, we need to gain knowledge about the effects of space stressors on the onset and evolution of serious diseases.

It is also important to unravel the role of gravity in wound and trauma healing, tissue regeneration, remodelling and development. In all these processes mechanical factors play important, but still not completely known, roles. Due to unloading conditions, the space environment is particularly suitable for these studies^48^.

A better understanding of the scientific problems described above, could not only help manage a number of diseases both in space and on Earth, but it should also enable crew members to be better fitted to return to a gravity environment like on Earth or when arriving on Mars.

Last but not least, cancer is caused by an accumulation of genetic mutations^49^. Its onset and progression also depend on alterations of the microenvironment^50,51^. Cancer onset could be caused by chronic exposure to environmental stressors as encountered during long-term space missions^52^. Identifying mechanisms of cancer development and progression during spaceflights is crucial. Since the impairment of immune system activity plays an important role in cancer progression, and a healthy immune system can counteract tumor growth^53^, it would be interesting to elucidate how altered gravity may affect the existing balance between immunity and immune escape mechanisms in cancer. Moreover, the role played by the cancer microenvironment, namely the stromal components and the extracellular matrix, should be further investigated.

The development of countermeasures for the different space stressors is a fundamental aspect in the preparation of future space exploration missions. Before application, identified countermeasures must be tested and validated to know their effectiveness and possible side effects when applied during spaceflight or in suitable space analogs.

When physical countermeasures (exercise, physical therapies) are considered, investigations on the mechanisms underlying their therapeutic effects carried out in unloading conditions will be very important to elucidate any difference in comparison with their application in loading conditions^54^.

Drug treatments are also included among the countermeasures to prevent or counteract the effects induced by spaceflight. However, due to the pathophysiological adaptation occurring during spaceflight, the absorption, distribution, metabolism and elimination of drugs is expected to be different in space. The recent episode on the ISS in which the administration of anticoagulants became necessary to treat a case of asymptomatic venous thrombosis (VT) of the internal jugular vein of an astronaut, further highlights the importance of studying the pharmacodynamics/kinetics of drugs in space^55^. Therefore, assessing gravity-induced changes in the pharmacodynamics/kinetics of drugs is certainly a topic that requires many research efforts^56^.

## Proposed research activities

**Fig. 1:**
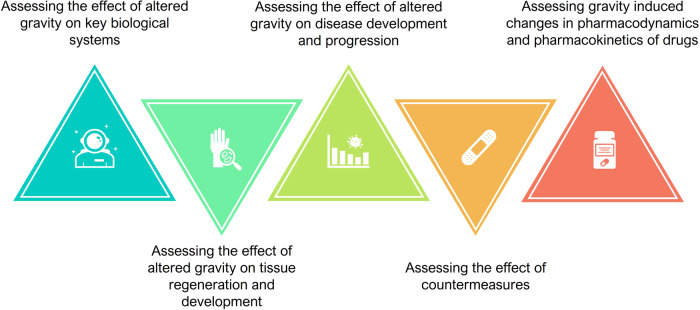
How to fill knowledge gaps. Main issues regarding how gravity alterations affect animal and human systems at a cellular/tissue level.

Summarizing what has been reported in the previous paragraph, five main scientific questions that are partially/totally unanswered have been identified (Fig. 1):

1. What are the effects of altered gravity on key biological systems such as the immune, musculoskeletal, cardiovascular, neurosensory, neuroendocrine, excretory, respiratory, and integumentary systems as well as on their metabolism and homeostasis?
2. What are the effects of altered gravity on tissue regeneration and development as well as on tissue engineering technologies?
3. What are the effects of altered gravity on the development and progression of diseases (e.g., cardiovascular and metabolic diseases, autoimmune diseases and allergies, cancer, genetic predisposition).
4. What is the effectiveness of countermeasures (e.g., physical countermeasures, nutrition, pharmaceuticals, nanoparticles and nanomaterials, other therapeutics) and what are the possible side effects when the countermeasures are applied during spaceflight?
5. What are the changes in pharmacodynamics/kinetics of drugs when these are used by crew members during spaceflight?

To answer these questions, a corresponding number of research lines should be implemented, with activities including both on ground and in-flight experiments, using all the available platforms and spanning from short to long timeline.

In more detail, the first research line should concern in-depth studies on effects of long-term exposure to spaceflight in order to understand basic processes in life sciences, and being able to support plans for long-duration and exploration missions (moon, Mars).

Studies should be designed with a dual purpose: i) on one side, evaluating the sum of multiple effects due to contemporary exposure to altered gravity, radiation, isolation, confinement, and psychophysical stress, which result in contemporary alterations in different organs and functions. ii) on the other side, trying to untangle the confounding effects of multiple stressors of the space exposome and to define the causes and contributing causes triggering different biological responses to help uncover the reasons and sequences of (mal)adaptations to space and place them in the context of human evolution. Moreover, a better understanding of the relationships between causes and effects is of crucial importance to implement effective countermeasures.

Indeed, basic research and human subjects´ research experiments should be bridged in the effort to connect biology, physiology and evolutionary anthropology, obtaining a more holistic approach to the process of human adaptation to space. Furthermore, the role of sex/gender in the biological response to spaceflight should be investigated more thoroughly.

Studies involving animal models can help explore the role of gravity with respect to spaceflight-related phenomena, like accelerated ageing, metabolic disorders or more social and behavioral-related phenomena. Animal models could also be applied to develop and test long duration Space Exploration related countermeasures such as in-flight artificial gravity, hibernation related protocols, nanotechnology, physical therapies, drugs, and probiotics. Small animal models (vertebrates like mice, fish, amphibians, other rodents or invertebrates like fruit flies, planarians, *C. elegans*, leeches and rotifers) could be of particular interest to study processes, functions and morphologies that have been conserved throughout evolution.

To heighten studies with animal models, in particular with rodents, access of European scientists to rodent research opportunities should be entertained either via collaboration with ISS partners for use of their facilities and/or via a close collaboration and use of the Italian Mouse Drawer System (MDS). Furthermore, when organizing animal/rodent studies both in-flight (altered gravity) or on the ground (unloading conditions, 1 *g* and hyper-*g*) a comprehensive tissue sharing program should be implemented.

Whichever model is used, it is recommended to consider the following key points when designing the experiments:

- The spaceflight exposure time, namely the duration of the experiments, should be increased, even to the length of the life cycle of the organism chosen as a model. An extended time exposure helps understand the risks associated to long duration missions;
- Higher statistical significance and reproducibility are needed to obtain “more quantitative” results. Therefore, the number of samples/subjects involved in the experiments should increase.
- Real time analysis and monitoring is important to avoid bias due to the return to Earth of the samples. Therefore, the development of suitable devices is needed;
- A highly integrated approach should be anticipated to make optimal use of the animal models (e.g. with elaborate Tissue Sharing Program).

A second research line should be addressed to explore and monitor the role of gravity (and the consequences of its alteration) in fundamental biological processes, such as growth and development, differentiation and organ morphogenesis, wound healing, tissue regeneration and restoration of homeostasis. Since the fine regulation of these processes is crucial for the development of organisms, their reaction to injuries and survival, a better knowledge of these topics in space environment is essential in view of future interplanetary missions, which will require long stay of crew members in space. In this perspective, research activities aimed at investigating the impact of spaceflight on reproduction, embryonic development, and multi-generational exposure should be increasingly carried out. Furthermore, the role of nutrition in growth, development, and response to injuries during spaceflight should be examined.

Beyond the investigation on embryogenesis in the space environment, research should be extended to other 3/4D biological processes, such as the growth and maturation of tissue constructs. Biotechnologies focused on tissue regeneration and engineering, not only have been indicated as enabling technologies for interplanetary missions, but they could also play a crucial role in coping with serious diseases on Earth. In this perspective, the identification of key omics-signatures for potential therapeutic applications both on Earth and long-term spaceflights would be extremely important.

One of the most important problems in programming crewed exploration missions beyond LEO is the increase in health risks due to prolonged exposure to radiation and microgravity. The condition of isolation and confinement and the prolonged psychophysical stress worsen the overall picture. Therefore, it is mandatory to implement a third line of research focused on the on-set, development and progression of serious acute and chronic diseases possibly induced by spaceflight. The identification of key omics-signatures of cancer and other diseases, such as metabolic and cardiovascular pathologies could be very important for their prevention and treatment.

It is well known that the cell microenvironment has a crucial role in cancer evolution, while experiments carried out in real and modeled microgravity demonstrated that extracellular matrix (ECM) properties can change in unloading conditions. Therefore, the cancer-associated microenvironment should be carefully studied in space. As regards the mechanisms underlying metabolic and cardiovascular diseases, studies should focus on low grade inflammation and mitochondrial dysfunction, which often characterize these kind of diseases.

A fourth line of research should focus efforts on the development of countermeasures enabling, from a biomedical point of view, future interplanetary missions. Protective (preventative) and therapeutic strategies based on both pharmacological and physical therapies should be implemented. The mechanisms of action of the countermeasures must be studied in the space environment, or by using facilities that model the space exposome, because differences are expected in comparison to what has been observed on Earth. Studies to elucidate the mechanisms of action of the countermeasures, evaluate their effectiveness, and to validate them, might be conducted using 2D and 3D in vitro models, ex vivo tissue models, and animal models, and then combined with studies on human subjects in space to confirm the results obtained with the models. Also, research on drug vectors (including nanomaterials) for medical applications in space is needed.

An important aspect to consider when specifically evaluating pharmacological treatments for use during spaceflight, is related to possible changes in drug pharmacodynamics/pharmacokinetics due to changes in human physiology induced by space adaptation mechanisms. Indeed, these changes could induce differences in absorption, distribution, metabolism and elimination of drugs. Studies carried out in rodents have reported that microgravity induces alterations in liver metabolism, causing a pathophysiological state similar to fatty liver disease, a condition that can affect the processes of absorption, distribution, metabolism, and excretion of drugs^57,58^. Therefore, a fifth research line should be specifically aimed at: i) evaluating the effectiveness of drugs in spaceflight conditions; ii) investigating the possible changes induced by the space environment in the action mechanisms of drugs and possible adverse reactions; iii) defining treatment protocols and dosages for use during spaceflight. Studies in spaceflight conditions should be systematically compared to suitable ground-controls in order to estimate the impact of spaceflight conditions on drug effects, drug metabolism, side effects, drug toxicity and adverse reactions.

The most relevant knowledge gaps and activities are reported in Table 1.

**Table 1:** Recommendations in short (3 years), middle (6 years) and long term (> 10 years).

Open fundamental scientific questions	Proposed Research Activities including ground & space experiments	Suitable testbed environment (Ground, LEO, BLEO, Moon, Mars,)	Space relevance (importance of altered gravity and/or relevance for space exploration)	Timeline
1: Assessing the effect of altered gravity on key biological systems such as the immune, musculoskeletal, cardiovascular, neurosensory, endocrine, excretory, respiratory and integumentary systems, their metabolism and homeostasis. It is also important to investigate the effect of gravitational alterations on functions and processes of outmost importance, as well as their pathological implications, such as coagulation, acute and chronic inflammation, antibody affinity maturation and its genetic machinery, stromal function and activation, metabolic pathways, temperature and its control. Studying the long-term consequences of the above effects (risk of development and progression of acute and chronic diseases)	Studying the effects of long-term exposure to spaceflight, in order to understand basic processes in life sciences and being able to support plan long-duration and exploration missions (Moon, Mars). Evaluating the sum of multiple effects due to the contemporary exposure to altered gravity, radiation, isolation, confinement and psychophysical stress, which result in contemporary alterations in different organs and functions. Connecting biology, physiology and evolutionary anthropology: a more holistic approach, human scoped experiments but also basic research studies are needed to help uncover causes of (mal)adaptations to space, placed in the context of human evolution. By using small animal models (vertebrates like mice, fish, amphibians, other rodents or invertebrates like fruit flies, planarians, C. elegans, leeches) study processes, functions and morphologies that are conserved throughout evolution. Animal models should be applied to explore the role of gravity or the lack thereof with respect to spaceflight related phenomena like increased ageing, metabolic disorders or more social and behavioral related phenomena. Such animal models could also be applied to develop long duration Space Exploration related countermeasure such as in-flight artificial gravity, hibernation related protocols, nanotechnology, physical therapies, drugs and probiotics. Access of European scientists as Project Coordinator for rodent research opportunities should be entertained either via collaboration with ISS partners for use of their facilities and/or via a close collaboration and use of the Italian Mouse Drawer System (MDS). For animal / rodent studies both in-flight (altered gravity) or ground based (unloading conditions, 1 g and hyper-g) a comprehensive tissue sharing program should be implemented. For all models: a) The spaceflight exposure time, that is the duration of the experiments involving the above models, should be increased, even to the length of the life cycle of the organism. b) Higher statistical significance and reproducibility are needed to obtain “more quantitative” results. c) Real time analysis and monitoring are important to avoid bias due to the return to Earth of the samples. d) A highly integrated approach should be anticipated to make optimal use of the animals (e.g. with elaborate Tissue Sharing Program). Investigating the role of sex/gender on the biological response to spaceflight. Examining whether the biological response to spaceflight is “spaceflight” vs. the built environment (e.g. altered gravity/radiation vs. hardware/closed system).	Ground-based (micro- and partial gravity simulation and hypergravity) in vitro and ex vivo models in/beyond LEO Animal models in LEO Animal models beyond LEO Humans in LEO Humans beyond LEO	Use of spaceflight environment for basic and applied research: Extended microgravity to permit the effect of sub g accelerations to be explored Ground based studies can explore response in microgravity analogue or hypergravity and gravity transitions Space Exploration relevance: Understanding the effect of spaceflight on medically relevant cells, tissues and organ systems and the development of countermeasures. Animal models enable research that is not possible with cellular systems or human subjects	Short Medium
2. Assessing the effect of altered gravity on tissue regeneration and development	Investigating and monitoring the fundamental processes of organ morphogenesis, regeneration, differentiation, and homeostasis under altered gravity. Identify key omics-signatures for potential therapeutic application on Earth and long-term spaceflights. Examine the impact of spaceflight on reproduction and multi-generational exposure. Examine the impact of spaceflight on embryonic development and other 3/4D biological processes. Examine the role of nutrition in the biological response to spaceflight.	Ground (micro- and partial gravity simulation and hypergravity). in vitro and ex vivo models in/beyond LEO Animal models in LEO Animal Models beyond LEO Humans in LEO Humans beyond LEO	Use of the spaceflight environment for basic and applied research: Increase understanding of how decreased mechanical load can impact tissue regeneration. Space Exploration relevance: Understanding the effect of spaceflight on medically relevant biological processes and the development of countermeasures.	Medium
3. Assessing the effect of altered gravity on disease development and progression	Identify key omics-signatures of cancer and other diseases’ development and progression. Analysis of changes in the cancer-associated microenvironment. Analysis of the role of age in the response to spaceflight-induced changes. Understanding thrombosis development and resolution.	Ground in vitro and ex vivo models in/beyond LEO Animal models in LEO Animal Models beyond LEO Humans in LEO Humans beyond LEO	Use of the spaceflight environment for basic and applied research: Increased understanding of how decreased mechanical load impacts disease onset and progression. Increase understanding of how spaceflight impacts disease onset and progression to better inform health risks for participants. Space Exploration relevance: Understanding the effects of spaceflight on medically relevant biological processes, risk assessment and the development of countermeasures.	Medium
4. Assessing the effect of countermeasures	Identification of adequate countermeasures, including physical countermeasures. Evaluation and validation of these countermeasures on in vitro 3D models, ex vivo tissue models and in vivo animal models in space is needed to confirm the protective or therapeutic effect, combined with studies on human subjects in space to confirm the results obtained with models. Using the above models, identify the most suitable drug candidates and/or vectors (including nanomaterials) for medical applications in space, as protective or therapeutic countermeasures against different space stressors (radiation, altered gravity, confinement, etc.).	Ground-based (micro- and partial gravity simulation and hyper- gravity) in vitro and in vivo models in/beyond LEO Animal models in LEO Animal models beyond LEO Humans in LEO Humans beyond LEO	Use of the spaceflight environment for basic and applied research: Evaluation of novel therapeutics for spaceflight. Space Exploration relevance: Understanding the effects of spaceflight on medically relevant biological processes, risk assessment and the development of countermeasures.	Medium
5. Assessing gravity-induced changes in the pharmacodynamics/kinetics of drug treatments. The mechanisms underlying the therapeutic effects of physical therapies should also be investigated in unloading conditions to elucidate any difference in comparison with loading conditions.	Confirm that they are still active under spaceflight conditions and maintain their therapeutic and health properties. Identify the factors and extent to which gravity may affect the active properties of the drug treatments. Identify possible changes to the adverse drug reaction profile of drug treatments. Conduct health tests on in vitro 3D models, ex vivo tissue models and in vivo animal models in space (and/or isolation, confinement) to confirm the protective or therapeutic effect. The results should be systematically compared to suitable ground-controls in order to estimate the impact of spaceflight conditions, combined with studies on human subjects in space to confirm the results obtained with models.	Ground-based (micro- and partial gravity simulation and hyper- gravity) in vitro and in vivo models in/beyond LEO Animal models in LEO Animal models beyond LEO Humans in LEO Humans beyond LEO	Use of the spaceflight environment for basic and applied research: Increase understanding of the impact of altered spaceflight physiology on pharmacodynamics. Increase understanding of the impact of spaceflight on drug shelf-life and kinetics. Space Exploration relevance: Understanding the effects of spaceflight on medically relevant biological processes, risk assessment and the development of countermeasures.	Medium Long

On-ground research activities require the use of microgravity analogs and microgravity simulators. Ground-based microgravity analogs include long-duration bed-rest and water immersion. The ground-based microgravity simulators include the following: clinostat, random positioning machine (RPM), rotating wall vessel (RWV) or Rotary Cell Culture System (RCCS) and Magnetic Levitation (ML)^59^. While microgravity analogs replicate some microgravity-induced physiological responses, microgravity simulators are platforms that can be used to model the exposure of cells, biological tissue samples and small animal models to microgravity conditions. Since conducting experiments in space is not always a viable option, ground-based microgravity analogs and simulators are widely used in space biology/physiology studies. Moreover, also on ground analogues such as submarines and Concordia Station in Antarctica are used to study the effects of isolation and confinement on human physiology. Thus, it is necessary to use these facilities coherently and to be careful when comparing results obtained by using different platforms^60^.

## Priorities for the ESA space programme (altered gravity and/or Exploration relevance)

The results of the above research activities will contribute to understand the mechanisms underlying temporary functional alterations in the human organism during spaceflight, their causes, recovery timelines and effects possibly leading to health risks and a progression towards chronic diseases.

The results will contribute to implementing preventative and therapeutic countermeasures as well as diagnostic tools to monitor astronauts’ health, manage health risks and supply effective therapies and health care facilities in future long-lasting space exploration missions. With these results, novel and original therapeutic solutions for spaceflight could also be found.

It is very important to highlight that these studies also offer the possibility of investigating topics of basic and applied research. The extended microgravity exposure will allow to explore the effect of sub *g* accelerations and make a comparison with the response in microgravity analogue or hypergravity and gravity transitions explored by ground-based studies. Moreover, it will be possible to increase the understanding of the impact of spaceflight on drug shelf-life and kinetics and to evaluate the effect of spaceflight-induced physiological alterations on pharmacodynamics.

The space environment offers a unique opportunity to study the role of gravity and mechanical factors on biological processes. By carrying out these studies, a better understanding of how decreased mechanical load can impact fundamental processes as tissue regeneration or disease onset and progression, could be reached. The perspectives of human exploration of the moon and Mars will need to further expand the knowledge on G-level transitions and especially the effects of life under at 1/6 or 1/3 of Earth gravity.

## Benefit for earth and industrial relevance

As above reported, many pathophysiological changes observed during and after spaceflight can represent models of a series of chronic diseases commonly observed on Earth, mostly affecting the elderly and frail subjects. The possibility to conduct studies on pathophysiological alterations in healthy and well-characterized subjects in space can shed new light on the cellular and molecular mechanisms underlying chronic diseases, help to find therapeutic targets and implement effective countermeasures to prevent the onset of these diseases or slow their progression, and, potentially increase our understanding of the molecular basis of frailty.

## Conclusions

In conclusion, for planning future manned interplanetary missions, it is necessary to expand our knowledge about the effects induced in human and animal organisms by the different stressors of the space exposome, namely microgravity, radiation, confinement, isolation and psychophysical stress. It is also required to have a greater awareness of the long-term consequences that could occur at a systemic level due to the multiple and concomitant alterations due to adaptation to spaceflight. Efforts should be made to progress in the implementation of effective countermeasures to monitor astronaut’s health as well as to prevent and treat diseases occurring during spaceflight. The application of advanced technologies (nanotechnologies, 3D printing, tissue engineering and regeneration, etc.) has a crucial role in the implementation of the research programs recommended in the roadmap.

### Reporting summary

Further information on research design is available in the Nature Research Reporting Summary linked to this article.

### Supplementary information

Reporting Summary
